# Laparoscopic Versus Open Cholecystectomy: A Prospective Matched-Cohort Study

**DOI:** 10.1155/1996/32413

**Published:** 1996

**Authors:** Robert J. Porte, Bas C. De Vries

**Affiliations:** Department of Surgery Westeinde Hospital The Hague The Netherlands

## Abstract

To compare the results of laparoscopic cholecystectomy (LC) and open cholecystectomy (OC) for symptomatic cholelithiasis in elective surgery we performed a prospective matched-cohort study. Hundred consecutive patients who underwent LC in the period Sept. 1990-June 1992, and 100 patients who were age and sex matched and underwent an elective OC in the foregoing two years (1989-1990) were studied. The median operation time for LC (75, 40-180 min) was significantly longer than for OC (55, 20-155 min; p < 0.001). Postoperative hospitalization was significantly shorter after LC (3, 1-16 days), compared with OC (7, 4-22 days; p<0.001). Conversion of LC to OC occurred in 12 (12%) patients initially scheduled to undergo LC. Complications occurred in 5 patients (5%) after LC and in 5 patients (5%) after OC. The calculated expenses (operation and postoperative hospitalization, 3rd class) were approximately fl. 3740,- for LC (excl. investments for pieces of apparatus) and fl. 6725,- for OC. This study demonstrates that LC can be performed safely with the number of complications comparable to those for OC. Bile duct injury is a serious potential threat. The main advantages ofLC are the minimal trauma, with more rapid recovery. Insurers seem to benefit from reduced postoperative disability and earlier discharge.


*HPB Surgery*, 1996, Vol. 9, pp. 71–75
Reprints available directly from the publisher
Photocopying permitted by license only

© 1996 OPA (Overseas Publishers Association)
Amsterdam B.V. Published in The Netherlands
by Harwood Academic Publishers GmbH
Printed in Malaysia

### Laparoscopic Versus Open Cholecystectomy: A Prospective Matched-Cohort Study

ROBERT J. PORTE and BAS C. DE VRIES

Department of Surgery, Westeinde Hospital, The Hague, The Netherlands

*(Received October 28, 1993)*

To compare the results of laparoscopic cholecystectomy (LC) and open cholecystectomy (OC) for symptomatic cholelithiasis in elective surgery we performed a prospective matched-cohort study. Hundred consecutive patients who underwent LC in the period Sept. 1990–June 1992, and 100 patients who were age and sex matched and underwent an elective OC in the foregoing two years (1989–1990) were studied. The median operation time for LC (75, 40–180 min) was significantly longer than for OC (55, 20–155 min; $p<0.001$). Postoperative hospitalization was significantly shorter after LC (3, 1–16 days), compared with OC (7, 4–22 days; $p<0.001$). Conversion of LC to OC occurred in 12 (12%) patients initially scheduled to undergo LC. Complications occurred in 5 patients (5%) after LC and in 5 patients (5%) after OC. The calculated expenses (operation and postoperative hospitalization, 3rd class) were approximately fl. 3740,- for LC (excl. investments for pieces of apparatus) and fl. 6725,- for OC. This study demonstrates that LC can be performed safely with the number of complications comparable to those for OC. Bile duct injury is a serious potential threat. The main advantages of LC are the minimal trauma, with more rapid recovery. Insurers seem to benefit from reduced postoperative disability and earlier discharge.

KEY WORDS: Laparoscopic cholecystectomy open cholecystectomy complications costs

### INTRODUCTION

The introduction of laparoscopic cholecystectomy (LC) has been a significant milestone in the treatment of gallstone disease. By this method the gallbladder is removed through four small incisions using laparoscopic techniques. Since its introduction in France in 1989[4], it has rapidly become available in the rest of Europe and the United States. In many centers LC is now the treatment of choice for symptomatic cholelithiasis[1,3,8,16,18]. Although the effectiveness of this technique has been suggested by several studies, the number of clinical trials comparing LC with the "gold standard", the traditional open cholecystectomy (OC), are sparse. The earliest reports have stressed the potential advantages of LC, as early recovery, shorter hospital stay and less pulmonary impairment[5,6,15,17]. However, more recent reports warn of the higher risk of common bile duct injuries that may be associated with LC[14,19].

To establish the advantages and potential risks of LC, studies comparing this technique with the OC are necessary. However, after the widespread introduction and acceptance of LC among the more educated and demanding patient population it has become practically impossible to perform prospective randomised trials.

In our hospital, data on diagnosis, treatment and complications of each patient are prospectively collected and stored in a computerized data base. We used these prospectively collected data to compare the results of LC over a 2-year period and the results of OC performed in a foregoing 2-year period. Studied were differences in operation time, postoperative hospitalization, complications and costs.

*Address for correspondence*: R. J. Porte, Dept. of Surgery, Leiden University Hospital, P.O. Box 9600, 2300 RC Leiden, The Netherlands.

### PATIENTS AND METHODS

All operations were performed under general anaesthesia and all patients received prophylactic antibiotics and subcutaneous heparin during the perioperative period.

### Laparoscopic Cholecystectomy

The first LC was performed in our hospital in September 1990. During the period September 1990–June 1992, 100 patients were considered for undergoing a LC. The indication for LC was symptomatic cholelithiasis, without signs of cholangitis, acute cholecystitis or choledocholithiasis. Obesity or a history of upper-abdominal surgery were not considered contraindications for LC. The diagnosis symptomatic cholelithiasis was confirmed by ultrasonographic investigation in all cases.

LC was performed by a standardized procedure[11]. After the cyctic artery and cystic duct were identified, they were clipped and transsected. The gallbladder was dissected by electrocautery and removed through the small umbilical wound. All wounds were closed with 1 or 2 fascia sutures and some skin sutures or steristrips.

### Open Cholecystectomy

A control group was constituted of patients who underwent an OC in a 2-year period (1989–1990), preceding the introduction of LC in our hospital. During this period 238 patients underwent OC. This was on an elective basis in 196 cases and an acute procedure in 42 cases. In the group of 196 elective procedures exploration of the common bile duct or another coincidental (abdominal) surgical procedure was necessary in 10 patients. These patients were excluded from the study. Of the remaining 186 cases, 100 patients were age and sex matched with the LC group.

A traditional OC was performed through a right subcostal incision. All data were prospectively collected and obtained form a computerized data base.

### Statistics

Data were subjected to computerized analysis (PATFILE statistical package). Continuous variables are given as median (range) and were analyzed by a nonparametric test for independent variables, the Mann-whitney test. Values for $p < 0.05$ were considered to be significant.

### RESULTS

Since the OC-group was matched for age and sex with the LC-group, these data were comparable for both groups. The median (range) age in the LC-group was 43 (18–88) years and 43 (20–79) years in the OC-group. The male/female ratio was 19/81% for both groups.

Data of all patients was analyzed according to the intention-to-treat principle, which means that patients who were scheduled to undergo LC but instead underwent OC for any reason, remained in the LC group.

### Conversion of Laparoscopic to Open Cholecystectomy

In our hospital a very liberal policy regarding the indication for conversion is used. Unnecessary risks are avoided and laparotomy is performed in all cases in which the anatomy is unclear or complications, that cannot be controlled laparoscopically, occur.

Conversion of LC to OC occured in 12 (12%) of the 100 patients initially scheduled to undergo LC. The reasons for conversion to laparotomy included difficult dissection due to adhesions after previous upper-abdominal operations or chronic cholecystitis (five patients), a stone in the cystic duct (two patients) and leakage of bile or bleeding (two patients). Two other patients required laparotomy because the gallbladder or stones were lost in the abdominal cavity and could not be found by laparoscopy. The latter is currently no longer considered as an indication for laparotomy since it has become clear that these stone are not harmfull and do not cause problems postoperatively. In one morbidly obese patient laparotomy was needed because the trocars were to short to pass the pannus and enter the abdominal cavity.

### Operation Time

The median operation time for LC was significantly longer than for OC. The distribution of operation time among both groups is depicted in Figure 1. The median (range) operation time for LC was 75 (40–180) min and 55 (20–155) min for OC ($p < 0.001$). During the study period operation time for LC showed a tendency to become shorter.

### Hospitalization Period

The median postoperative hospitalization period for LC was significantly shorter than for OC. The

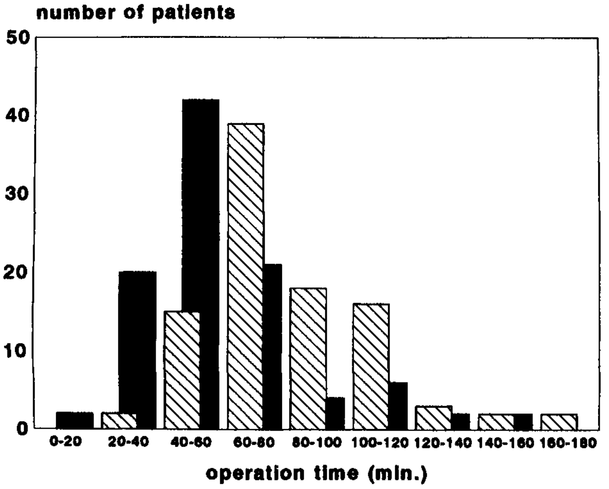

**Figure 1** Distribution of operation time in patients undergoing laparoscopic (n = 100; hatched bars) and open cholecyctectomy (n = 100; black bars).

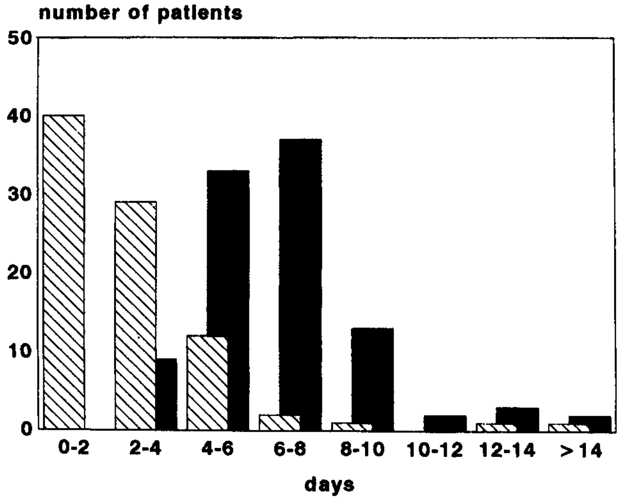

**Figure 2** Distribution of postoperative hospitalization time in patients undergoing laparoscopic (n = 100; hatched bars) and open cholecyctectomy (n = 100; black bars).

distribution of postoperative hospitalization time among both groups is presented in Figure 2. The median postoperative hospital stay was 3 (1–16) days after LC and 7 (4–22) days after OC ($P < 0.001$).

### Complications

Complications after LC and OC are shown in Table 1. Complications were seen in 5 of the 100 patients in both groups. In one patient 5 days after LC it appeared that the common bile duct had been partially clipped accidently. This patient became icteric post-operatively and an endoscopic retrograde cholangiopancreaticography (ERCP) demonstrated the stenosis. Laparotomy was necessary to remove the clip and regenerate bile flow. This patient recovered completely without any further surgery. There were no deaths in OC group, whereas one elderly patient died after LC due to a myocardial infarction.

**Table 1** Complications after laparoscopic and open cholecystectomy

| *Complication* | *Laparoscopic Cholecystectomy (N = 100)* | *Open Cholecyctectomy (N = 100)* |
|---|---|---|
| Wound infection | 2 | 2 |
| Bleeding (postop.) | – | 1 |
| Urinary tract infection | – | 2 |
| Bile duct injury | 1 | – |
| Biliary pancreatitis | 1 | – |
| Lost instrument | 1 | – |
| Total | 5 (5%) | 5 (5%) |

### Financial Analysis

In the Netherlands different reimbursement systems are used for patients insured by the National Health Insurance and by private insurance companies. For patients insured by the National Health Insurance there is usually a standard fee for each diagnostic or therapeutic procedure and hospitalization day. For example, the standard fee for a cholecystectomy is independent of the number of disposable instruments used during the operation. The reduced hospitalization time made LC less expensive than OC for the National Health Insurance (Table 2). However the hospital expenses for LC are greater because they include the use of disposable instruments and investments for new instruments as well (Table 2).

**Table 2** Financial analysis of laparoscopic and open cholecystectomy

| *Costs per patient* | *Laparoscopic cholecystectomy* | *Open cholecystectomy* |
|---|---|---|
| National Health Insurance | | |
| Hospitalization | Fl. 2475 | Fl. 5775 |
| Operation | Fl. 1265 | Fl. 950 |
| Total | Fl. 3740 | Fl. 6725 |
| Expenses for disposable instruments | Fl. 505 | nihil |

Not included are investments for new pieces of apparatus.

### DISCUSSION

Since its introduction, LC has been performed with increasing frequency. The cosmetic advantages of LC are obvious. Many studies have documented that LC is associated with less postoperative pain, a shorter hospital stay, and a more rapid recovery and return to normal activity than OC[5,6,15,17]. However, most reports were based on open studies and uncontrolled series of LC. In some studies the results of LC were compared with the results of OC, available from other reports[3,8,18]. The efficiency and safety of any new medical procedure, however, should be established by comparing it with the currently available and generally accepted technique. Symptomatic cholelithiasis has been treated by OC with excellent results for more than hundred years. The currently acceptable mortality rate (0.2%) and complication rates (3%–5%) for selective OC are quite low[9,10]. Randomised clinical trials comparing LC and OC have become almost impossible due to the rapid acceptance and increasing popularity of LC. Objective analysis of the results and especially complications, however, remains warranted.

We compared the results of LC and OC in patients referred from the same population to one general hospital. Patient groups were formed prospectively. Potential bias by differences in age and sex were excluded by matching both groups for those two variables.

Conversion of LC to OC occurred in 12% of the patients. This is comparable with a series reported by Grace *et al.*[6], who did 6 laparotomies in a series of 50 patients (12%) who underwent LC for both acute and chronic gallbladder disease. Others have reported conversion rates that are somewhat lower (3%–5.3%)[3,8,15,18]. This difference can undoubtedly be ascribed to our liberal policy for conversion and the learning curve with the procedure. In our opinion, conversion should never be seen as a complication or failure. Hesitation to perform a laparotomy when this is indicated and inevitable, may lead to serious and irreversible morbidity. For this reason, it is important that patients are always informed preoperatively about the possibility of conversion.

Complications were seen in 5% of the patients in both groups, which is comparable to the complication rates published by others. In different studies complications were seen in 1.6%–6% of the patient[3,8,15,19]. In our series, most complications could be qualified as minor. A serious complication occurred in one patient with an accidental partial occlusion of the common bile duct by a clip. Following the initial enthousiastic reports on LC, later studies have stressed the risk of increased number of complications, especially common bile duct injuries[14,19]. It has become clear that common bile duct injuries are a potential threat in LC. The reported incidence of bile duct injuries for OC ranges from 0%–0.5%[5,7]. The incidence of common bile duct injury in laparoscopic series ranges from 0–3%[1,3,13]. Several investigators have recommended routine operative cholangiography to define more clearly the common bile duct anatomy and thereby reduce the risk of bile duct injury[2,12]. Currently, there are no convincing data that operative cholangiography should be performed routinely as part of LC. In fact, we did not use intraoperative cholangiography in any patient. At this point in the development of LC the incidence of bile duct injury appears to correlate more closely with the operative experience of the surgeon than with a particular policy towards operative cholangiography.

The decreased length of hospitalization associated with LC has been considered to be one of its main benefits. In our series we found a median postoperative hospital stay of 3 days compared to 7 days after OC. These data are comparable to those published by others[3,6,15]. The faster recovery and earlier discharge resulted in a reduction in costs for patients insured by the National Health Insurance. Others have also reported lower costs for patients undergoing LC in Ireland[6]. This situation may be completely different in other countries. Indeed, in the U.S.A. higher expenses have been reported for LC than for OC[15]. The true costs for the hospital, however, are also higher in our situation, due to the higher operating room expenses, including longer duration of the operation and use of costly equipment needed for this procedure, such as disposable trocars and clip appliers. This should also be considered when evaluating the financial aspects of this new technique.

This study demonstrates that LC can be performed safely with the number of complications comparable to those for conventional, open cholecystectomy. Bile duct injury is a serious potential threat. The main advantages of LC are the minimal trauma, with more rapid recovery. Insurers seem to benefit from reduced postoperative disability and earlier discharge. Although LC is becoming the treatment of choice for symptomatic cholelithiasis, continued study and long-term follow ups are needed to evaluate and further develop this relatively young surgical technique. Besides proper training of unexperienced surgeons, adequate education of endoscopic techniques, both in experimental situations and *in vivo*, should be part of training programs for surgical residents.

### REFERENCES

1. Berci, G., Sackier, J. M. (1991) The Los Angeles experience with laparoscopic cholecystectomy. *Am. J. Surg.*, **161**, 382–384.
2. Berci, G., Sackier, J. M., Paz-Partlow, M. (1991) Routine or selected intraoperative cholangiography during laparoscopic cholecystectomy? *Am. J. Surg.*, **161**, 355–360.
3. Cuschieri, A., Dubois, F., Mouiel, J., *et al.* (1991) The European experience with laparoscopic cholecystectomy. *Am. J. Surg.*, **161**, 385–387.
4. Dubois, F., Berthelots, G., Levard, H. (1989) Cholecystectomy par coelioscopie. *Presse. Med.*, **18**, 980.
5. Gilliland, T. M., Traverso, L. W. (1990) Modern standards for comparison of cholecystectomy with alternative treatments for symptomatic cholelithiasis with emphais on long term relief of symptoms. *Surg. Gynecol. Obstet.*, **170**, 39–44.
6. Grace, P. A., Quereshi, A., Coleman, J., *et al.* (1991) Reduced postoperative hospitalization after laparoscopic cholecystectomy. *Br. J. Surg.*, **78**, 160–162.
7. Hermann, R. E. (1976) A plea for safer technique of cholecystectomy. *Surgery*, **79**, 609–611.
8. Larson, G. M., Vitale, G. C., Casey, J., *et al.* (1992) Multipractice analysis of laparoscopic cholecyctectomy in 1,983 patients. *Am. J. Surg.*, **163**, 221–226.
9. McSherry, C. K. (1991) Cholecystectomy: the gold standard. *Am. J. Surg.*, **158**, 174–178.
10. Morgenstern, L., Wong, L., Berco, G. (1992) Twelve hundred open cholecystectomies before the laparascopic era. A standard for comparison. *Arch. Surg.*, **127**, 400–403.
11. Olsen, D. O. (1991) Laparoscopic cholecystectomy. *Am. J. Surg.*, **161**, 339–344.
12. Petelin, J. B. (1991) Laparoscopic approach to common duct pathology. *Surgical Laparoscopy and Endoscopy*, **1**, 33–41.
13. Reddick, E. J., Olsen, D., Spaw, W., *et al.* (1991) Safe performance of difficult laparoscopic cholecystectomies. *Am. J. Surg.*, **161**, 377–381.
14. Rossi R. J., Schirmer W. J., Braasch, J. W., *et al.* (1992) Laparoscopic bile duct injuries. Risk factors, recognition, and repair. *Arch. Surg.*, **127**, 596–602.
15. Stoker, M. E., Vose, J., O'Mara, P., Maini B. S. (1992) Laparoscopic cholecystectomy. A clinical and financial analysis of 280 operations. *Arch. Surg.*, **127**, 589–595.
16. The Southern Surgeons Club. (1991) A prospective analysis of 1518 laparoscopic cholecystectomies. *N. Engl. J. Med.*, **324**, 1073–1078.
17. Vitale, G. C., Collet, D., Larson, G. M., *et al.* (1991) Interruption of professional and home activity after laparoscopic cholecystectomy among French and American patients. *Am. J. Surg.*, **161**, 396–398.
18. Voyles, C. R., Petro, A. B., Meena, A. L., *et al.* (1991) A practical approach to laparoscopic cholecystectomy. *Am. J. Surg.*, **161**, 365–370.
19. Wolfe, B. M., Gardiner, B. N., Leary, B. F., Frey, C. F. (1991) Endoscopic cholecystectomy. An analysis of complications. *Arch. Surg.*, **126**, 1192–1198.

